# Cortical spectral matching and shape and volume analysis of the fetal brain pre- and post-fetal surgery for spina bifida: a retrospective study

**DOI:** 10.1007/s00234-021-02725-8

**Published:** 2021-05-01

**Authors:** Nada Mufti, Michael Aertsen, Michael Ebner, Lucas Fidon, Premal Patel, Muhamad Bin Abdul Rahman, Yannick Brackenier, Gregor Ekart, Virginia Fernandez, Tom Vercauteren, Sebastien Ourselin, Dominic Thomson, Luc De Catte, Philippe Demaerel, Jan Deprest, Anna L. David, Andrew Melbourne

**Affiliations:** 1 Elizabeth Garrett Anderson Institute for Women’s Health, University College London, 1st Floor Charles Bell House, 43-45 Foley Street, W1W 7TS London, UK; 2 School of Biomedical Engineering & Imaging Sciences (BMEIS), King’s College London, London, UK; 3 Department of Radiology, University Hospitals Katholieke Universiteit (KU), Leuven, Belgium; 4 Medical Physics and Biomedical Engineering, University College London, London, UK; 5 Radiology Department, Great Ormond Street Hospital for Children, London, UK; 6 Paediatric Neurosurgery Department, Great Ormond Street Hospital for Children, London, UK; 7 Department of Obstetrics and Gynaecology, University Hospitals, Katholieke Universiteit (KU) Leuven, Leuven, Belgium; 8 Cluster ‘Women and Child’, Dept. Development and Regeneration, Biomedical Sciences, Katholieke Universiteit (KU) Leuven, Leuven, Belgium

**Keywords:** Myelomeningocele, Fetal surgery, MRI, Shape, Volume, Cortical spectral matching

## Abstract

**Purpose:**

A retrospective study was performed to study the effect of fetal surgery on brain development measured by MRI in fetuses with myelomeningocele (MMC).

**Methods:**

MRI scans of 12 MMC fetuses before and after surgery were compared to 24 age-matched controls without central nervous system abnormalities. An automated super-resolution reconstruction technique generated isotropic brain volumes to mitigate 2D MRI fetal motion artefact. Unmyelinated white matter, cerebellum and ventricles were automatically segmented, and cerebral volume, shape and cortical folding were thereafter quantified. Biometric measures were calculated for cerebellar herniation level (CHL), clivus-supraocciput angle (CSO), transverse cerebellar diameter (TCD) and ventricular width (VW). Shape index (SI), a mathematical marker of gyrification, was derived. We compared cerebral volume, surface area and SI before and after MMC fetal surgery versus controls. We additionally identified any relationship between these outcomes and biometric measurements.

**Results:**

MMC ventricular volume/week (mm^3^/week) increased after fetal surgery (median: 3699, interquartile range (IQR): 1651–5395) compared to controls (median: 648, IQR: 371–896); *P* = 0.015. The MMC SI is higher pre-operatively in all cerebral lobes in comparison to that in controls. Change in SI/week in MMC fetuses was higher in the left temporal lobe (median: 0.039, IQR: 0.021–0.054), left parietal lobe (median: 0.032, IQR: 0.023–0.039) and right occipital lobe (median: 0.027, IQR: 0.019–0.040) versus controls (*P* = 0.002 to 0.005). Ventricular volume (mm^3^) and VW (mm) (*r*=0.64), cerebellar volume and TCD (*r* = 0.56) were moderately correlated.

**Conclusions:**

Following fetal myelomeningocele repair, brain volume, shape and SI were significantly different from normal in most cerebral layers. Morphological brain changes after fetal surgery are not limited to hindbrain herniation reversal. These findings may have neurocognitive outcome implications and require further evaluation.

## Introduction

Myelomeningocele (MMC) is the most frequent form of open neural tube defect and results in abnormal development of the terminal spinal cord and associated meninges entailing an extrusion of the spinal cord into a cerebro-spinal fluid (CSF)–filled sac [1, 2]. In addition to motor and urological disability, children with MMC exhibit varying degrees of impaired cognitive performance [3]. The reasons for neurocognitive impairment are incompletely understood. The constellation of brain changes comprising the Chiari II malformation (CIIM) which include small posterior fossa, caudal displacement of hindbrain structures and supratentorial anomalies [4, 5] may be partly responsible either directly or by causing hydrocephalus through abnormal CSF circulation [6, 7]. Hydrocephalus requires treatment with ventriculoperitoneal shunts in up to 80% of cases and causes mechanical stretching of brain parenchyma leading to abnormal unmyelinated white matter development which can be illustrated in diffusion tensor imaging (DTI) studies [8–15]. This neural tract damage can predispose to supratentorial anomalies such as altered gyrification patterns (e.g., polymicrogyria) and periventricular nodular heterotopia [8–15]. In MMC children who have undergone postnatal closure, gyrification measured by magnetic resonance imaging (MRI) correlates with motor and cognitive function [9]. Mechanical compression of the cerebellum may also lead to ischaemic changes compounding these deficiencies [8–10, 14].

A landmark RCT (Management of Myelomeningocele Study, MOMS) provided level 1 evidence that fetal surgical MMC closure can reduce ventriculoperitoneal shunt requirement by 40% and hindbrain herniation at 12 months [6, 7]. It has been suggested that fetal surgery might also improve cognitive and motor outcomes in MMC infants. It is unclear to what extent such benefits are related to CIIM reversal and halting progression of hydrocephalus [14], or whether the surgery confers additional benefits to the developing brain.

A better understanding of the impact of fetal MMC closure might help our understanding of mechanisms underlying cognitive impairment in this group of patients and help with prognostication [3, 9, 14]. Advanced MRI techniques are now available to measure volume of unmyelinated white matter, ventricles and cerebellum for analysis, whilst simultaneously reducing impact of fetal motion. We used a novel automated technique to reconstruct the fetal brain and quantitatively studied longitudinal cortical gyrification using markers of surface curvature, before and after MMC fetal surgical repair.

## Methods

This was a single-centre retrospective case control study approved by the Ethics Committee for clinical research of Universitair Ziekenhuis (UZ) Leuven (S60814). Funders had no direction in study design, data collection, data analysis, manuscript preparation or publication decision. The data was uploaded via a secure cloud platform which ensures complete anonymisation prior to transfer using XNAT technology [16].

### Dataset and fetal imaging

Twelve fetuses with MMC were evaluated with MRI before and after fetal surgery. These were compared to 12 ‘early’ and 12 ‘late’ gestational age-matched control fetuses that had been assessed for other congenital and placental anomalies but no central nervous system (CNS) anomaly. The eligibility criteria for fetal surgery were those used in the MOMS trial [6, 17]. In our cohort, the lesion level started no higher than T12 and was no lower than S1 with hindbrain herniation present. The corpus callosum was normal and present in all cases, but three, whereby the splenium appeared to be either thin or absent. In all cases, the mesencephalic aqueduct was noted to be patent before and after surgery. All MRI was performed at UZ Leuven Hospital, Belgium, with the same acquisition protocol. The images were acquired on a 1.5 T MR system (Aera, Siemens, Erlangen, Germany) without maternal or fetal sedation. Two small body coils were placed adjacent to each other on the maternal abdomen, with the mother in either the supine or the left lateral decubitus position. T2-weighted single-shot fast spin-echo (SSFSE) was performed on the fetal brain in multiple orientations with at least one stack in axial, coronal and sagittal planes. The sequence was performed using slice thickness of 3 mm, 1000 ms repetition time, 133 ms echo time and voxel size of 0.6 × 0.6 × 3.0 mm. Other sequences performed during this routine clinical protocol included echo planar imaging, T1-weighted imaging and diffusion-weighted imaging with total acquisition time of 40 min.

### Automated super-resolution reconstruction

**Fig. 1:**
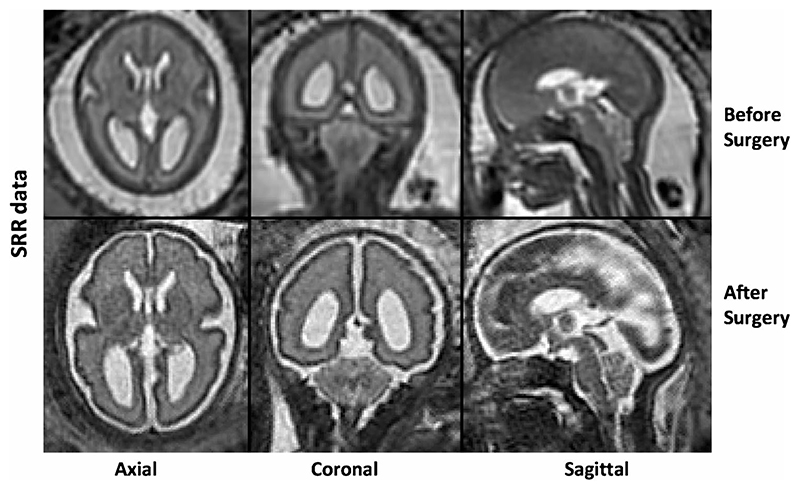
An example of the original super-resolution reconstruction (SRR) data displayed pre-operatively (top row) and post-operatively (bottom row) in the axial, coronal and sagittal planes (right to left)

A novel automated SRR algorithm was used to reconstruct an isotropic 3D volume of the fetal brain using the native T2-weighted 2D MRI stacks. This involved automatic fetal brain localisation, segmentation and robust reconstruction [18]. At least three orthogonal T2-weighted image stacks, acquired in at least three orientations (i.e. axial, coronal, sagittal), were used for the algorithm in each case. The fetal brain was automatically segmented in each 2D stack followed by iterative motion correction and volumetric reconstruction steps. Iterative 3D reconstructions were estimated from motion-corrected slices using outlier-robust SRR methods to account for image artefacts and potential misalignments as part of the motion-correction step. All images were reconstructed at an isotropic resolution of 0.8 mm. Visualisation of the obtained 3D reconstruction in standard radiological anatomical planes was achieved by automatic alignment to a spatio-temporal atlas [19]. This reconstruction method was previously assessed systematically whereby experiments showed that each step of the pipeline outperformed state-of-the art methods in both segmentation and reconstruction comparisons which included expert-reader subjective quality assessments by two paediatric radiologists [18]. Prior to reaching our final dataset as described in the previous section, a total of seven cases (three MMC images before and after surgery, two ‘early’ and two ‘late’ controls) where excluded due to poor SRR quality. Figure 1 shows an example of the original data quality of an MMC case included in the final dataset.

### Manual-based and atlas-based segmentation

Brain masks for the pre-operative MMC group were resampled from their corresponding post-operative counterparts after affine alignment. Automatic segmentation of the unmyelinated white matter, ventricles and cerebellum was thereafter performed using template preterm neonatal brain and deep learning-based segmentations which provided good initial segmentation quality [20–22]. Unmyelinated white matter is defined as the brain parenchyma underneath the cortical plate and above the basal ganglia. We use this definition to include any other transitory cerebral structures which may be evident in this region, such as the subplate. Additionally, our automatic segmentations initially included all intracranial cerebro-spinal fluid (CSF). This was thereafter manually corrected to include only the ventricular system which included the lateral, third and fourth ventricle; the cerebral aqueduct; cavum septum pellucidum (CSP) and the cavum vergae (CV). In our MMC cohort, as demonstrated in previous literature, we have observed several cases where the CSP and CV were absent, and therefore, it was simpler at this current stage to include them in our ventricular parcellations [23]. All automatically segmented structures were manually corrected by a trained obstetrician (NM) and corrected as necessary thereafter by a consultant board-certified paediatric neuroradiologist (MA) after which meshes were generated using ITK-Snap™ [24]. This process of manual refinement was necessary due to the complexity of intracranial abnormalities seen in MMC, such as direct apposition of the cortex to the skull in Chiari II malformation, and inability to differentiate between structures especially if compressed in a small posterior fossa.

### Volumetric, shape and cortical spectral matching analysis

**Fig. 2:**
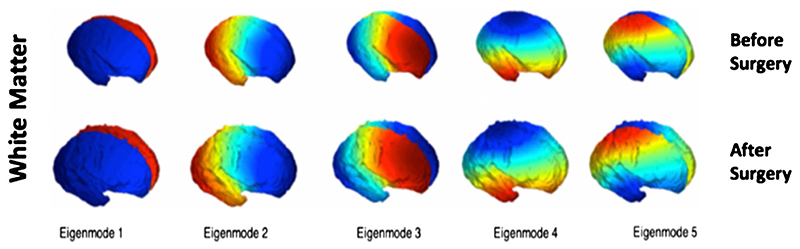
The first five spectral modes of the unmyelinated white matter of a fetus for two different time points: before surgery (top row) and after surgery (bottom row)

We carried out a hierarchical analysis of volume, followed by a global shape analysis (shape parameter), and a local surface-based shape analysis (joint spectral matching) on our meshes. The volume (mm^3^) of each cerebral structure was calculated as the sum of all voxels from each segmentation multiplied by the voxel dimension of the reconstructed volume. Surface area (mm^2^) was calculated as the sum of triangle areas that compose each mesh. A global shape parameter (mm^−1^) was defined as surface area/volume. As the fetal brain undergoes significant changes in cortical folding, we additionally aimed to quantify this locally using a surface-based spectral matching technique to find longitudinal correspondences of the unmyelinated white matter surface features before and after fetal surgery [20]. A rigid coherent point drift algorithm was applied to find an initial correspondence for the intrasubject cortical regions before and after surgery. Joint spectral matching (JSM) was then used to find the correspondence for the intrasubject cortical structures at these two different time points [20]. In JSM, a dual-layered graph is produced whereby layers correspond to the surface of the unmyelinated white matter before and after surgery. The correspondence links from the initial intrasubject matching, connecting both layers and producing a set of shared eigenmodes of the surfaces [25, 26]. Figure 2 displays the first five eigenmodes for unmyelinated white matter before and after fetal surgery.

**Fig. 3:**
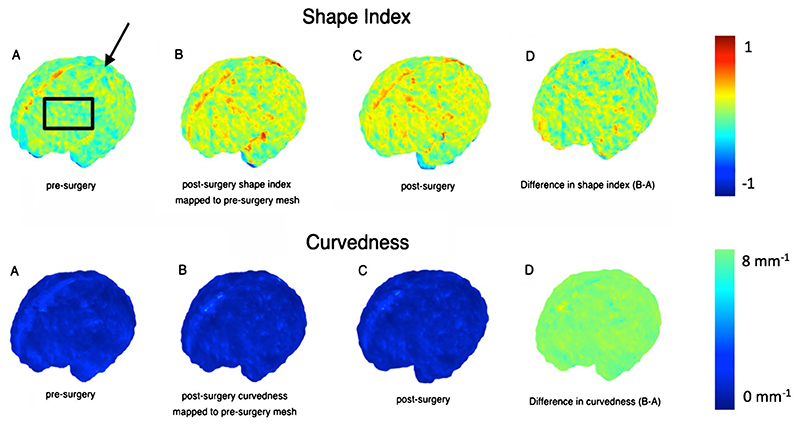
Map display (with accompanying colour scale (right)) of curvedness and shape index for the unmyelinated white matter of a fetus pre-surgery and post-surgery. **a** This map shows the apparent locations of development of several primary (black arrow) and secondary sulci (black box). **b** Post-surgery shape index mapped to the pre-surgery mesh. **c** Post-surgery mesh. **d** Difference in shape index and curvedness (B-A)

Although the meshes are quite different in the threedimensional space, with respect to different levels of folding, variation in shape, surface area and volume, they have similar representations in the spectral domain [26]. After mapping, the post-operative surface to the pre-operative counterpart using JSM, we computed the change in parameters at the vertex of each mesh to explore longitudinal gyri-fication. This is represented by mathematical markers of the curvature of the surface. Specifically, the curvedness (mm^-1^) expressing the extent of which a surface is curved, and the shape index (SI), defining the convexity of the surface [27]. SI therefore is a mathematical reference to the morphology of the external unmyelinated white matter surface acting as a surrogate measure of cortical folding. Figure 3 illustrates this concept whereby positive values are depicted in red/yellow and represent gyri (convex structures), and negative values in blue represent sulci (concave structures).

JSM allows mapping of curvedness and SI measurements from the post-op to the pre-op space. We furthermore separated the cortical surface into frontal, temporal, occipital and parietal lobes, and further divided them into the right and left cerebral hemispheres. This was performed by registering the corresponding gestation from the Boston fetal brain MRI atlas template [19] onto our unmyelinated white matter segmentations using non-rigid registration. Anatomical labels from the atlas were manually reorganised to represent the four brain lobes in the two hemispheres.

### Biometric measurements

Anatomical measurements were also performed on the SRR images, namely the transverse cerebellar diameter (TCD), ventricular width (VW), the cerebellar herniation level (CHL) and the clivus supraoccipital angle (CSO). These were selected as Aertsen et al. [28] demonstrated the reliability of these posterior fossae measurements on 2D MRI images as well as a significant change of these parameters within 1 week of prenatal surgery with a trajectory towards normal, the most evident of which is the CSO and CHL.

Both the TCD and VW were measured on the coronal plane at the level of the atria, whereby the VW was drawn on an axis perpendicular to that of the ventricle at mid-height of the atria [29]. The CHL was measured perpendicularly from the foramen magnum to the lowest cerebellar portion in the midsagittal plane [28]. Two lines were drawn along the clivus and the supraocciput, and the angle defined by their intersection was the CSO [30, 31]. The measurements were performed by two authors: NM, an obstetrician who was trained by PP, a consultant paediatric radiologist, on a training dataset. Correlation between these anatomical measurements and our volumetric and cortical spectral matching metrics was thereafter investigated.

### Statistics

Statistical analysis was performed in MATLAB (The Mathworks Inc., Natick, MA). Due to a small sample size and a non-normal distribution (as confirmed by the Anderson–Darling test), a Kruskal–Wallis H test was performed to compare mean differences between groups. Statistical significance was set below 5%. Correlations were assessed using Pearson’s correlation coefficient (*r*), where *r* < 0.3 indicated none or very weak correlation, 0.3 < *r* < 0.5 weak correlation, 0.5 < *r* < 0.7 moderate correlation and *r* > 0.7 strong correlation [32]. Only *r* values with moderate correlation are discussed.

## Results

### Demographics

Twelve MMC fetuses underwent MRI before (mean 23^+6^ ± 1^+0^ weeks + days, (range 22^+0^–25^+0^), and at an average of 2 weeks after open fetal surgery (mean 26^+1^ ± 1^+2^ weeks + days, (24^+0^–29^+0^)). The results were compared with measurements from two control groups of fetuses separated into two imaging time points: Early gestational age (GA) (*n* = 12, 23^+2^ ± 1^+2^ weeks + days, (21^+0^–25^+0^)) and a late GA (*n* = 12, 28^+6^ ± 1^+3^ weeks + days (26^+0^–30^+0^)). The control fetuses had normal CNS findings on ultrasound and underwent MRI for non-CNS indications (Online Resource Table 1). All control fetuses had normal CNS MRI findings. There was no difference in GA between MMC fetuses before fetal surgery and the early control group (*p* = 0.181). However, in the late GA group, control cases underwent fetal MRI at a significantly later GA than the fetal surgery cases, an average of 2 weeks + 4 days (*p* < 0.001, Online Resource Table 1).

### Volume and shape parameter

Cerebellar volume in MMC fetuses increased after surgery (median: 886 mm^3^, IQR: 29–1969 mm^3^), but significantly less so than in controls (median: 4226 mm^3^, IQR: 3123–4994 mm^3^), *p* < 0.001 (Online Resource Fig. 1 (c)). This difference however could be explained by the significantly later GA at MRI between MMC fetuses after surgery and the late control group. To account for the difference in GA, we therefore compared the paired rate of change in volume/week between the MMC and control fetuses, which was not significantly different (Online Resource Fig. 1 (d)). MMC cerebella however followed a similar trajectory of shape parameter change to controls after surgery, relative to their initial shape parameter (Online Resource Fig. 2 (a)).

**Fig. 4:**
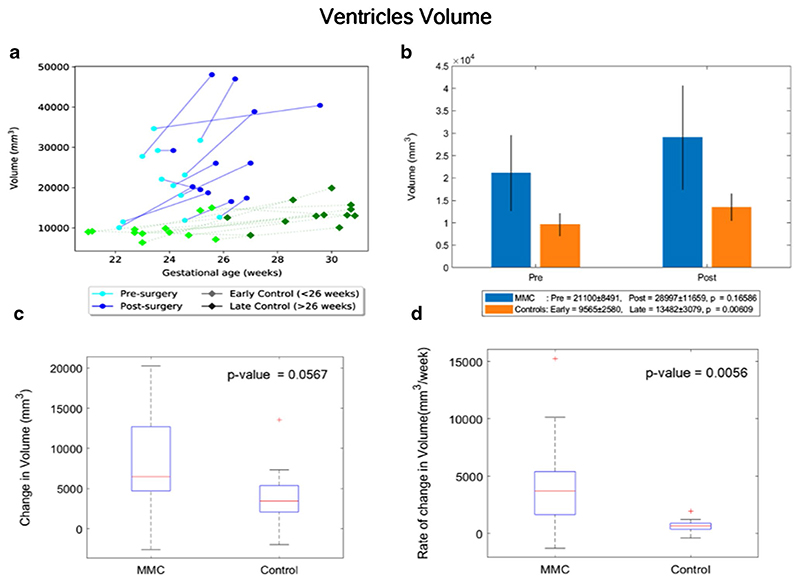
**a** Volume (mm^3^) of MMC ventricles pre-surgery and postsurgery, and age-matched controls against gestational age in weeks. **b** Volume (mm^3^) of MMC ventricles pre-surgery and post-surgery compared to early and late controls. The *p* values compare differences in measurements between pre-MMC surgery and post-MMC surgery, and early and late controls to controls. **c** Absolute difference in change in volume (mm^3^) of ventricles after surgery for MMC compared to controls. **đ** Rate of change in volume/time (mm^3^/week) of ventricles after surgery for MMC against age-matched controls

By contrast, the paired MMC ventricular volume/week increase, hence the CSF weekly increase rate, was significantly higher in MMC patients after surgery (median = 3699 mm^3^/week, IQR: 1651–5394 mm^3^/week), in comparison to controls (median = 648 mm^3^/week, IQR: 371–896 mm^3^/week), *p* = 0.006 (Fig. 4).

**Fig. 5:**
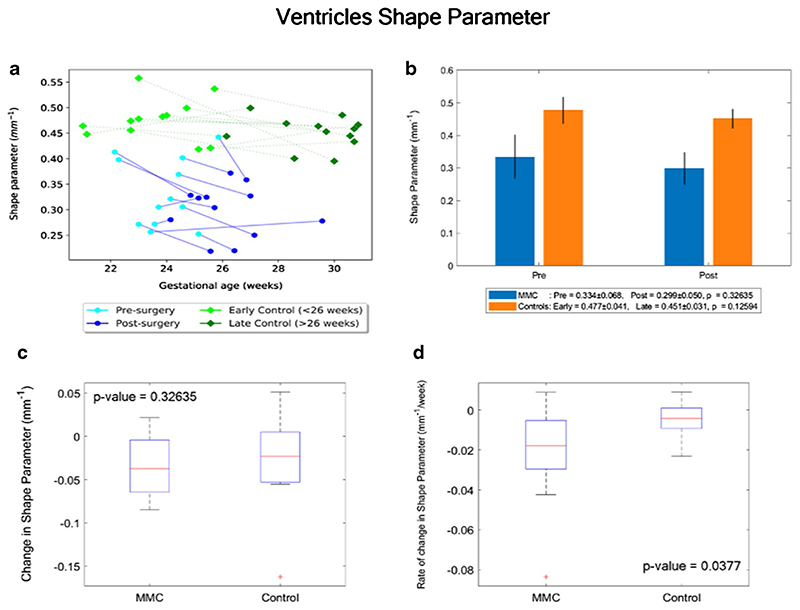
**a** Shape parameter (mm^−1^) of MMC ventricles pre-surgery and post-surgery, and age-matched controls against gestational age in weeks. **b** Shape parameter (mm^−1^) of MMC ventricles pre-surgery and post-surgery compared to early and late controls. The *p* values compare differences in measurements between pre-MMC surgery and post-MMC surgery, and early and late controls to controls. **c** Absolute difference in change in shape parameter (mm^−1^) of ventricles after surgery for MMC compared to controls. **d** Rate of change in shape parameter/time (mm^−1/^week) of ventricles after surgery for MMC against age-matched controls

Similarly, the paired change of ventricular shape parameter/week was significantly higher in MMC patients (median: − 0.018 mm^−1^/week, IQR: − 0.029– −0.005 mm^−1^/week), in comparison to controls (− 0.004 mm^−1^/week, IQR: − 0.009–0.001 mm^−1^/week), *p* = 0.038 (Fig. 5).

Unmyelinated white matter volume was increased in MMC fetuses after surgery compared to that before (median: 16,177 mm^3^, IQR: 9390–23,109 mm^3^), but this volume increase was less than in control fetuses (median: 44,527 mm^3^, IQR: 32,226–49,487 mm^3^), *p* = 0.001 (Online Resource Fig. 3 (c)). After accounting for the difference in GA of the later MRI imaging by calculating the paired change of unmyelinated white matter volume/week, we observed no significant difference between the two groups (Online Resource Fig. 3 (d)). MMC unmyelinated white matter shape parameter similarly exhibited a reduced change after surgery (median: − 0.008 mm^−1^, IQR: − 0.030–0.006 mm^−1^), in comparison to controls (median: − 0.027 mm^−1^, IQR: − 0.051–^−^0.009 mm^−1^), (Online Resource Fig. 4 (c)). There was however no significant difference in the paired shape parameter/week between the two groups (Online Resource Fig. 4 (d)).

### Shape index

**Fig. 6:**
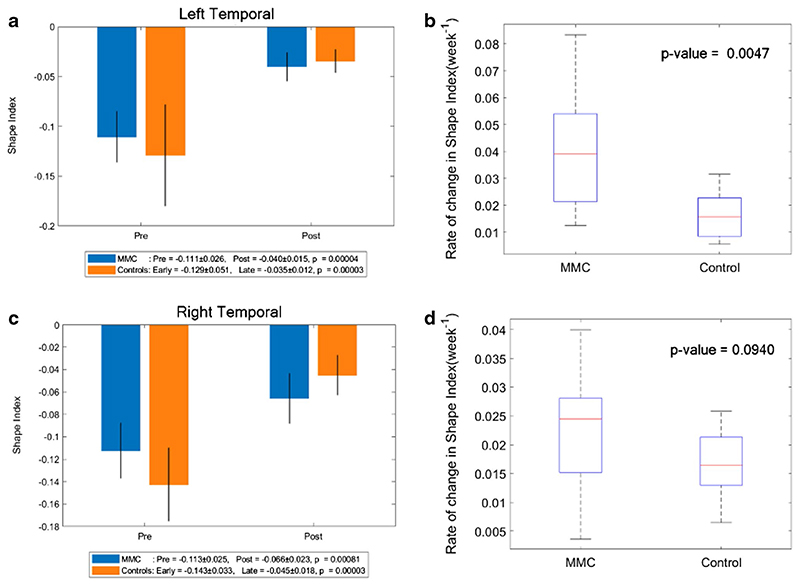
**a** Shape index of MMC left temporal lobe pre-surgery and post-surgery compared to early and late controls. The *p* values compare differences in measurements between pre-MMC surgery and post-MMC surgery, and early and late controls to controls. **b** Rate of change in shape index/time (week^−1^) of the left temporal lobe after surgery for MMC against age-matched controls. **c** Shape index of MMC right temporal lobe pre-surgery and post-surgery compared to early and late controls. The *p* values compare differences in measurements between pre-MMC surgery and post-MMC surgery, and early and late controls to controls. d Rate of change in shape index/time (week^−1^) of right temporal lobe after surgery for MMC against age-matched controls

**Fig. 7:**
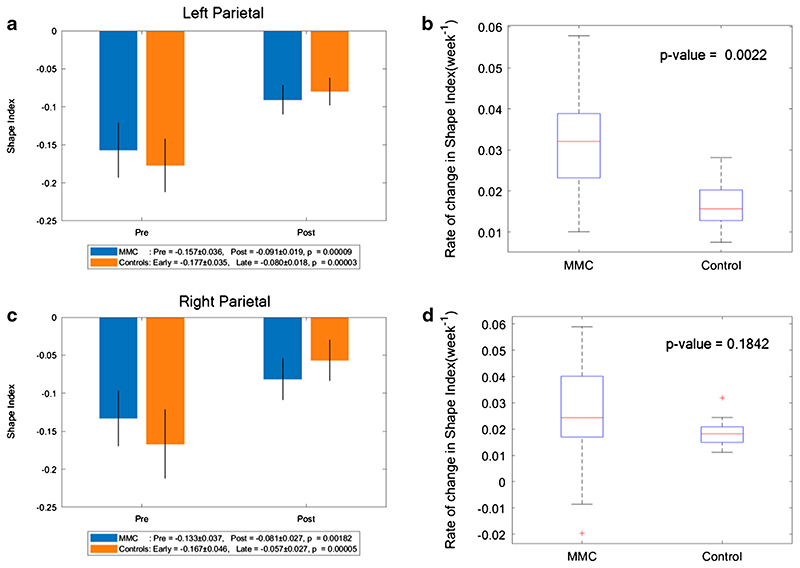
**a** Shape index of MMC left parietal lobe pre-surgery and post-surgery compared to early and late controls. The *p* values compare differences in measurements between pre-MMC surgery and post-MMC surgery, and early and late controls to controls. **b** Rate of change in shape index/time (week^−1^) of left parietal lobe after surgery for MMC against age-matched controls. **c** Shape index of MMC right parietal lobe pre-surgery and post-surgery compared to early and late controls. The *p* values compare differences in measurements between pre-MMC surgery and post-MMC surgery, and early and late controls to controls. **d** Rate of change in shape index/time (week^−1^) of right parietal lobe after surgery for MMC against age-matched controls

**Fig. 8:**
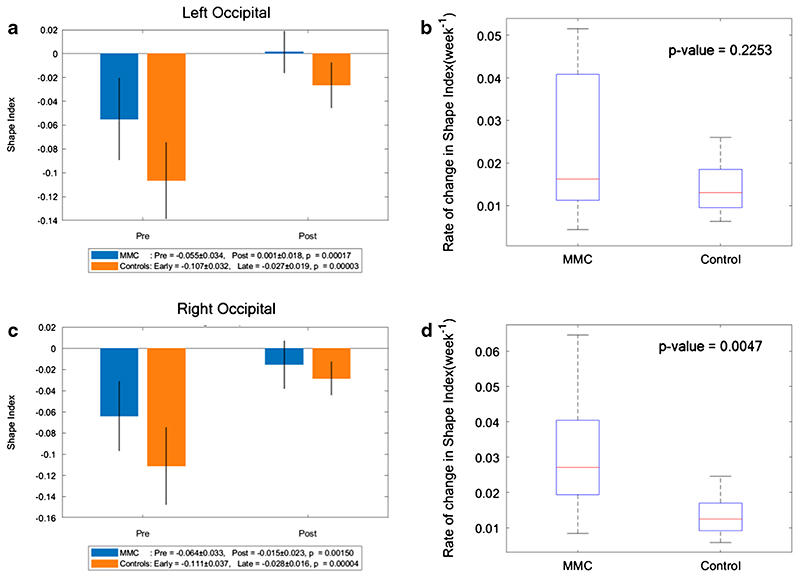
**a** Shape index of MMC left occipital lobe pre-surgery and post-surgery compared to early and late controls. The *p* values compare differences in measurements between pre-MMC surgery and post-MMC surgery, and early and late controls to controls. **b** Rate of change in shape index/time (week^−1^) of left occipital lobe after surgery for MMC against age-matched controls. **c** Shape index of MMC right occipital lobe pre-surgery and post-surgery compared to early and late controls. The *p* values compare differences in measurements between pre-MMC surgery and post-MMC surgery, and early and late controls to controls. **d** Rate of change in shape index/time (week^−1^) of the right occipital lobe after surgery for MMC against age-matched controls

A more positive SI demonstrates the appearance of gyri (convex structures) and has no units. The change in SI in unmyelinated white matter of MMC fetuses was significantly less after surgery (median: 0.011, IQR: − 0.002–0.025) in comparison to that of controls (median: 0.067, IQR: 0.058–0.075), *p* < 0.001 (Online Resource Fig. 5). When unmyelinated white matter was further separated into the frontal, temporal, occipital and parietal lobes, and divided into the right and left hemispheres, it was noticeable that the SI of MMC fetuses pre-surgery was higher in comparison to early controls in all areas (Online Resource Fig. 6, and Figs. 5, 6, 7). In order to account for a potential influence of GA difference at the later MRI scan, the paired change in average SI/week was compared. This showed a significant change in SI/week in the left temporal lobe (median: 0.039, IQR: 0.021–0.054; *p* = 0.005), left parietal lobe (median: 0.032, IQR: 0.023–0.039; *p*=0.002), and right occipital lobe (median: 0.027, IQR: 0.019–0.040; *p* = 0.005) with a trajectory similar to the control group (Figs. 6, 7, 8).

**Fig. 9:**
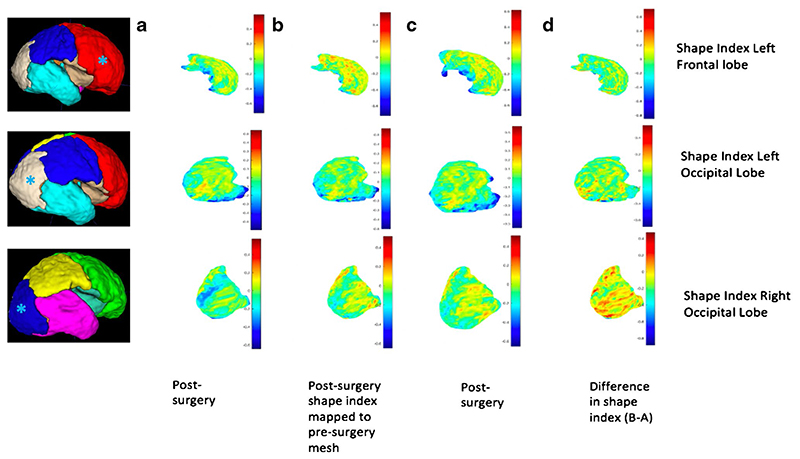
Map display of shape index for the left frontal lobe (top row), left occipital lobe (middle row) and right occipital lobe (bottom row) of a fetal mesh. (**a**) Pre-surgery, (**b**) post-surgery shape index mapped to the pre-surgery mesh, (**c**) post-surgery mesh and (**d**) difference in shape index and curvedness (B-A). For orientation purposes, a 3D brain mesh (right) is provided for the left frontal lobe (red), left occipital lobe (white) and right occipital lobe (dark blue). Asterisks (*) are provided on 3D brain meshes for lobe identification

The resultant post-operative SI was lower in comparison to late controls in all lobes, apart from the right frontal and occipital lobes. A visual representation of the SI for the left frontal and occipital lobes is shown in Fig. 9. A positive SI is depicted in red/yellow representing gyri (convex structures), and negative values are shown in blue to represent sulci (concave structures).

### Correlation of biometric measurements with shape, volumetric and SI analysis

There were no correlations, moderate or above, between the four anatomical markers we measured, namely CHL, TCD, VW and CSO, and unmyelinated white matter volume or shape parameter. There was a moderate positive correlation (*r* = 0.56) seen between cerebellar volume and TCD. Ventricular volume also showed moderate positive correlation with VW (*r*=0.64) (Online Resource Fig. 7). There were no correlations, moderate or above, between the four biometric measurements and unmyelinated white matter SI.

## Discussion

### Main findings

In this study, we have applied state-of-the art MRI technology to a unique application whereby we studied MMC cerebral development in the context of fetal surgery. We assessed changes of cerebellar volume and shape following fetal surgery in fetuses with MMC. We found that after accounting for differences in GA at time of MRI through calculating the paired change of volume and shape parameter/week, there were no significant differences when compared to GA-matched control fetuses without CNS abnormalities. However, we observed that MMC cerebella shape parameter changed in a trajectory similar to controls illustrating cerebellar herniation reversal.

We additionally illustrated that changes in MMC ventricular volume and shape parameter per week after surgery were significantly higher than those in control counterparts. By contrast, there was no change in unmyelinated white matter volume and shape parameter per week between MMC and GA-matched control fetuses. The difference however between MMC cortical SI before and after surgery was significantly less than controls. After parcellating unmyelinated white matter into two hemispheres and four lobes, we demonstrated that MMC cortical SI is higher across all regions pre-operatively in comparison to that in controls. Post-operatively, MMC cortical SI appeared to be less than controls in all lobes apart from the left frontal and occipital lobes. In an attempt to account for GA differences between groups, we studied change in SI per week which showed a significant higher increase in SI in the left temporal, left parietal and right occipital lobes. This translates into increased cortical surface convexities in comparison to controls. Lastly, we observed moderate positive correlation between VW and TCD and ventricular and cerebellar MMC growth, indicating how biometric measurements might be used in liaison with our volumetric, shape and surface analysis.

### Strengths and limitations

This study used novel MRI technology to permit volumetric and shape analysis in the MMC fetal brain, and further, to produce a mathematical representation of gyrification at the brain surface and map this to different regions of the MMC fetal brain. This has been demonstrated in children and young adults who had postnatal surgery in previous studies [8, 9]. Therefore, our techniques have adapted these observations to evaluate morphological changes in the fetal brain that occur in response to prenatal surgery in comparison with controls. To make this comparison, we assumed that a linear trajectory of growth, which although may be incorrect, has been suggested in previous MRI studies of fetal brain development [33–35]. In the absence of data with a higher temporal resolution, we feel that assuming a linear change in sulcation is appropriate. Of course, a higher-order model could be tested in the future with a larger dataset. One limitation of this study is that we used two groups of controls from different fetuses separated into early and late, to compare with MMC fetuses before and after surgery. This is due to the challenging nature of obtaining a group of controls with longitudinal data. Furthermore, to account for differences in GA between MMC fetuses post-surgery and late controls, we calculated change in volume, shape parameter and SI per week. The short interval period of MRI performed after prenatal surgery and lack of longitudinal antenatal imaging post-operatively is another limitation of this study but reflects practical difficulties in performing repeated prenatal MRI scans. Maturation of cortical gyrification is dependent on GA and is best seen on MRI at 30–32 weeks. By 34 weeks, all primary and some secondary sulci are visible on fetal MRI [36]. Thus, later imaging would provide further detail on development of cortical folding in MMC fetuses. Moreover, it would have been interesting to compare these results with fetuses eligible for prenatal surgery, but where parents have opted for postnatal therapy. This would provide a better indication of the effects of prenatal surgery on cortical development. A potential further consideration is that controls used for comparison with the MMC group had MRI due to other congenital fetal and placental abnormalities, and although none had demonstrable CNS anomalies, it could be argued that these cases do not represent normality.

### Interpretation

We have demonstrated no significant change in cerebellar volume growth per week of MMC fetuses after surgery compared to controls. This may be due to observing a period in fetal development when the cerebellum does not significantly increase in size in normal circumstances as there is known significant enlargement of the cerebellum postnatally [37]. Furthermore, previous studies have reported cerebellar mechanical compression caused by hindbrain herniation, which can lead to ischaemic changes and cerebellar dysgenesis [37]. This highlights the need for additional longitudinal antenatal imaging post-fetal MMC surgery to further investigate the development of cerebellar growth and shape change. We have additionally illustrated an improvement in cerebellar shape seen after surgery, with a trend towards a more normal growth trajectory which suggests a degree of reversibility of anomalous development due to fetal surgical intervention. The functional implications of this observation are currently unknown.

This study has additionally demonstrated that ventriculomegaly does not reduce once spinal closure is established after surgery. This is consistent with findings from other studies whereby obstructive ventricular widening was demonstrated 1 week after surgery [28]. The pathogenesis of ventriculomegaly is likely to be multifactorial, and we postulate that there may an impaired absorption of CSF back into the venous system. This may be due to a lag period, whilst absorption pathways mature in response to the increase CSF load post-repair. Therefore, monitoring the evolution of ventriculomegaly post-operatively remains essential to identify which cases may require neurosurgical intervention for hydrocephalus. In this current cohort, there is no postnatal or further antenatal follow-up data available to confirm our hypothesis. This motivates the need for a follow-up study with a larger sample size to investigate ventricular size post-surgery with additional longitudinal antenatal imaging.

Furthermore, this study showed no significant change in unmyelinated white matter growth and shape (surface area/volume) in fetuses after MMC surgery per week when compared to control fetuses with a normal CNS. However, some studies have reported features such as cortical thinning in children and adolescents who had postnatal surgery [9]. This has been attributed to gradual altered development of unmyelinated white matter pathways secondary to damaging mechanical forces from ventricular expansion and altered neuronal proliferation and migration [9, 38]. This emphasises the need for further longitudinal antenatal imaging post-fetal MMC surgery to investigate the development of unmyelinated white matter growth and shape change.

Finally, we sought to analyse the cortical surface after MMC surgery, in different parts of the fetal brain by using spectral matching. Cortical surface changes of our control fetuses relate to known patterns of cortical folding across GA [36, 39], and in this work, we illustrated observed differences in gyrification of the myelomeningocele fetal brain compared with that of a normal brain. We assumed a crosssectional representation of normal gyrification using our controls based on previous post-mortem histological and in vivo MRI evidence [35, 39]. We have been able to demonstrate that MMC cortical SI is higher pre-operatively in all regions of the brain in comparison to early controls. There was additionally a significant change in MMC SI/week observed in the left temporal, left parietal and right occipital lobes, with a resultant higher SI seen post-operatively in the left occipital lobe. Left to right brain asymmetry of the fetal brain has been previously described by advanced quantitative MRI studies, prompting speculation on functional and behavioural differences observed after birth [33, 39]. There is also some evidence of normal neurodevelopmental ‘torque’ in mid-gestation which may underlie some of the results that we observe [40]. These cortical surface changes may be multifactorial and not wholly influenced by the Chiari II malformation alone. There have been several hypotheses put forward on the processes that underlie normal gyrification and fetal brain such as genetic control, or abnormal white matter development and neural tract damage due to mechanical stretching from hydrocephalus in association with MMC [8–15, 35].

Furthermore, aberrant cortical folding in the myelomeningocele brain is in keeping with the findings of Treble [9] and Juranek et al. [8, 10] who identified abnormal cortical thickness and gyrification in children and adolescents with MMC in comparison to controls, whereby regions that were more gyrified or thicker tended to be negatively associated with cognitive and motor functions. More frequent and detailed antenatal longitudinal studies of different cortical folding patterns that we have seen in our fetal MMC brain cohort may be useful in interpreting the development of the fetal brain post-prenatal surgery and to investigate our observed gyrification asynchrony is preserved. This could reveal trajectories of development which could have precise functional predictions and improve case selection in the future. Studying aberrant folding in different cerebral regions could also be a promising tool in planning targeted treatment programmes such as speech, and physical therapy depending on which part of the brain is affected. Earlier focused rehabilitation in infancy is important as this is a critical time period of brain growth, maturation and intellectual development. The combination of increased plasticity of the infant brain and targeted therapy may offer a greater chance of improvement in cognitive and motor function [3, 14].

## Conclusion

This study applies novel MRI technology to demonstrate differences in volume, and shape of unmyelinated white matter, ventricles and cerebella before and after prenatal surgery for MMC compared to those of controls. Additionally, cortical surface matching analysis demonstrated a difference in the change in cortical surface convexities per week in the left temporal, left parietal and right occipital lobes compared to controls. It has been repeatedly demonstrated that fetal surgery for MMC results in improvement of hindbrain herniation. This study reinforces findings from neuroradiological literature suggesting that additional morphological changes (such as altered cortical folding) also occur in the supratentorial compartment which might have implications for neurocognitive outcome [41]. Further work utilising longitudinal imaging in late third trimester is critical taking into account the rapidly evolving MMC fetal brain. Moreover, correlation with other intracranial and extracranial anomalies such as callosal anomalies, and lesion level, may prove beneficial in improving our understanding of the MMC fetal brain development.

## Supplementary Material

The online version contains supplementary material available at https://doi.org/10.1007/s00234-021-02725-8.

Supplementary Information

## Data Availability

All data and material of this work are available on request from authors.

## Code availability

NiftyMIC is a research-focused toolkit for motion correction and volumetric image reconstruction of 2D ultra-fast MRI. It is available as on open-source on: https://github.com/gift-surg/NiftyMIC.
