# Supplementary material for: Cortical spectral matching and shape and volume analysis of the fetal brain pre- and post-fetal surgery for spina bifida: a retrospective study
Source: Neuroradiology. Author manuscript; Available in PMC 2022 Apr 1. (PMC8460513; doi:10.1007/s00234-021-02725-8)
Supplement: Supplementary Information [file EMS131081-supplement-Supplementary_Information.docx]

# Title:

Cortical Spectral Matching and Shape and Volume Analysis of the Fetal Brain Pre and Post Fetal Surgery for Spina Bifida: A Retrospective Study

# Authors:

Nada Mufti^1,3^, Michael Aertsen^2^, Michael Ebner^3,4^, Lucas Fidon^3^, Premal Patel^5^, Muhamad Bin Abdul Rahman^3^, Yannick Brackenier^3^, Gregor Ekart^3^, Virginia Fernandez^3^, Tom Vercauteren^3,4^, Sebastien Ourselin^3,4^, Dominic Thomson^6^, Luc De Catte^7^, Philippe Demaerel^2^, Jan Deprest^1,7^, Anna L David^1,7^, Andrew Melbourne^3,4^

# Affiliations:

1. Elizabeth Garrett Anderson Institute for Women’s Health, University College London, UK
2. Department of Radiology, University Hospitals Katholieke Universiteit (KU) Leuven, Belgium 3 School of Biomedical Engineering & Imaging Sciences (BMEIS), King’s College London, UK 4 Medical Physics and Biomedical Engineering, University College London, UK
3. Radiology Department, Great Ormond Street Hospital for Children, London, UK
4. Paediatric Neurosurgery Department, Great Ormond Street Hospital for Children, London, UK
5. Department of Obstetrics and Gynaecology, University Hospitals and cluster ‘Women and Child’, Dept. Development and Regeneration, Biomedical Sciences, Katholieke Universiteit (KU) Leuven, Leuven, Belgium

# Corresponding Author:

*Nada Mufti [n.mufti@ucl.ac.uk](mailto:n.mufti@ucl.ac.uk)

07515352229

1^st^ Floor Charles Bell House 43-45 Foley Street

London W1W 7TS

https://orcid.org/0000-0001-9839-8085


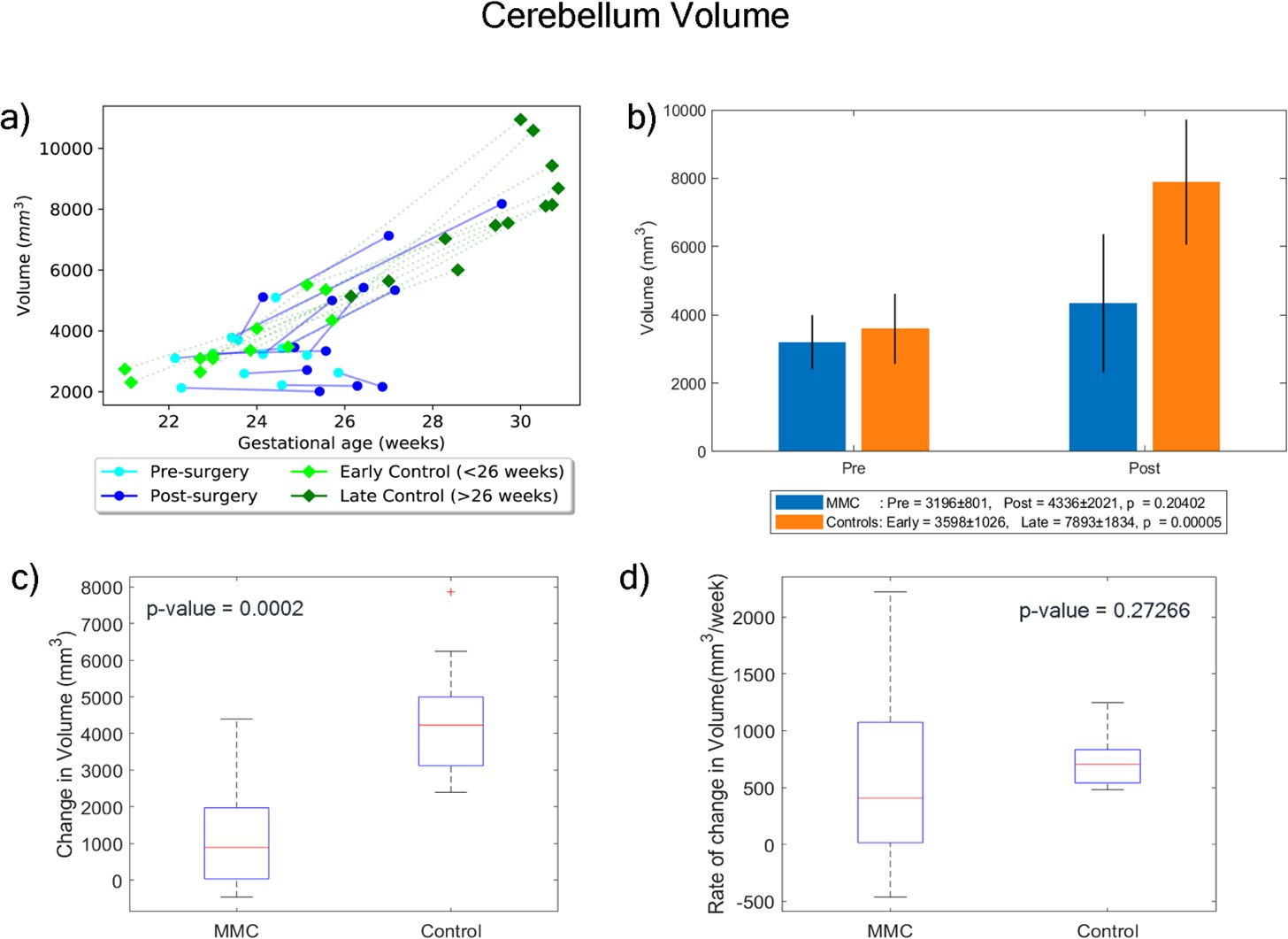


**Fig. 1** (a) Volume (mm^3^) of MMC cerebella pre and post-surgery, and age-matched controls against gestational age in weeks

(b) Volume (mm^3^) of MMC cerebella pre and post-surgery compared to early and late controls. The p values compare differences in measurements between pre and post MMC surgery, and early and late controls to controls (c) Absolute difference in change in volume (mm^3^) of cerebella after surgery for MMC compared to controls (d) Rate of change in volume/time (mm^3^/week) of cerebella after surgery for MMC against age-matched controls.


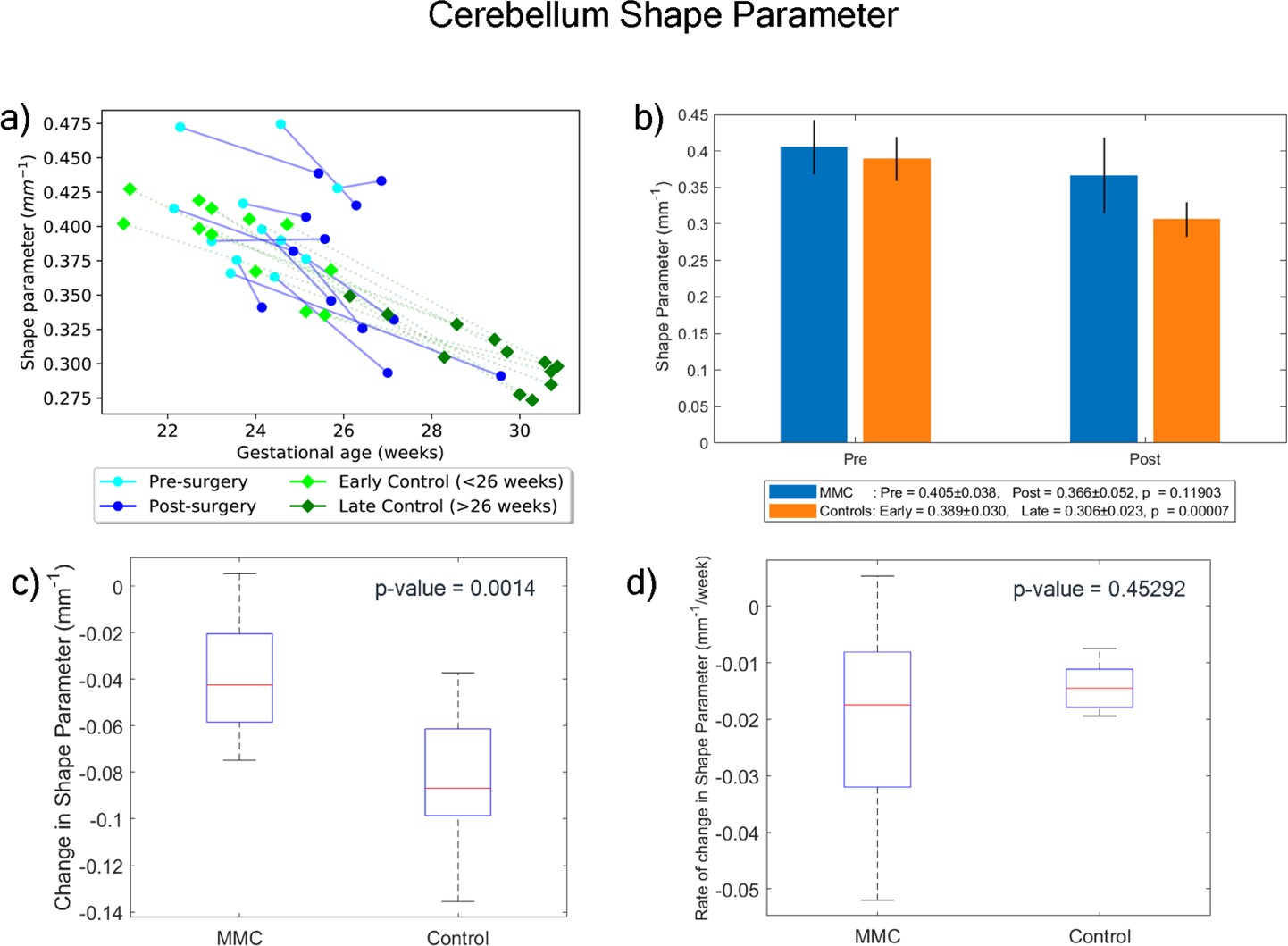


**Fig. 2** (a) Shape parameter (mm^-1^) of MMC cerebella pre and post-surgery, and age-matched controls against gestational age in weeks. (b) Shape parameter (mm^-1^) of MMC cerebella pre and post-surgery compared to early and late controls. The p values compare differences in measurements between pre and post MMC surgery, and early and late controls to controls (c) Absolute difference in change in Shape parameter (mm^-1^) of cerebella after surgery for MMC compared to controls (d) Rate of change in shape parameter/time (mm^-1/^week) of cerebella after surgery for MMC against age-matched controls.

Unmyelinated White Matter Volume


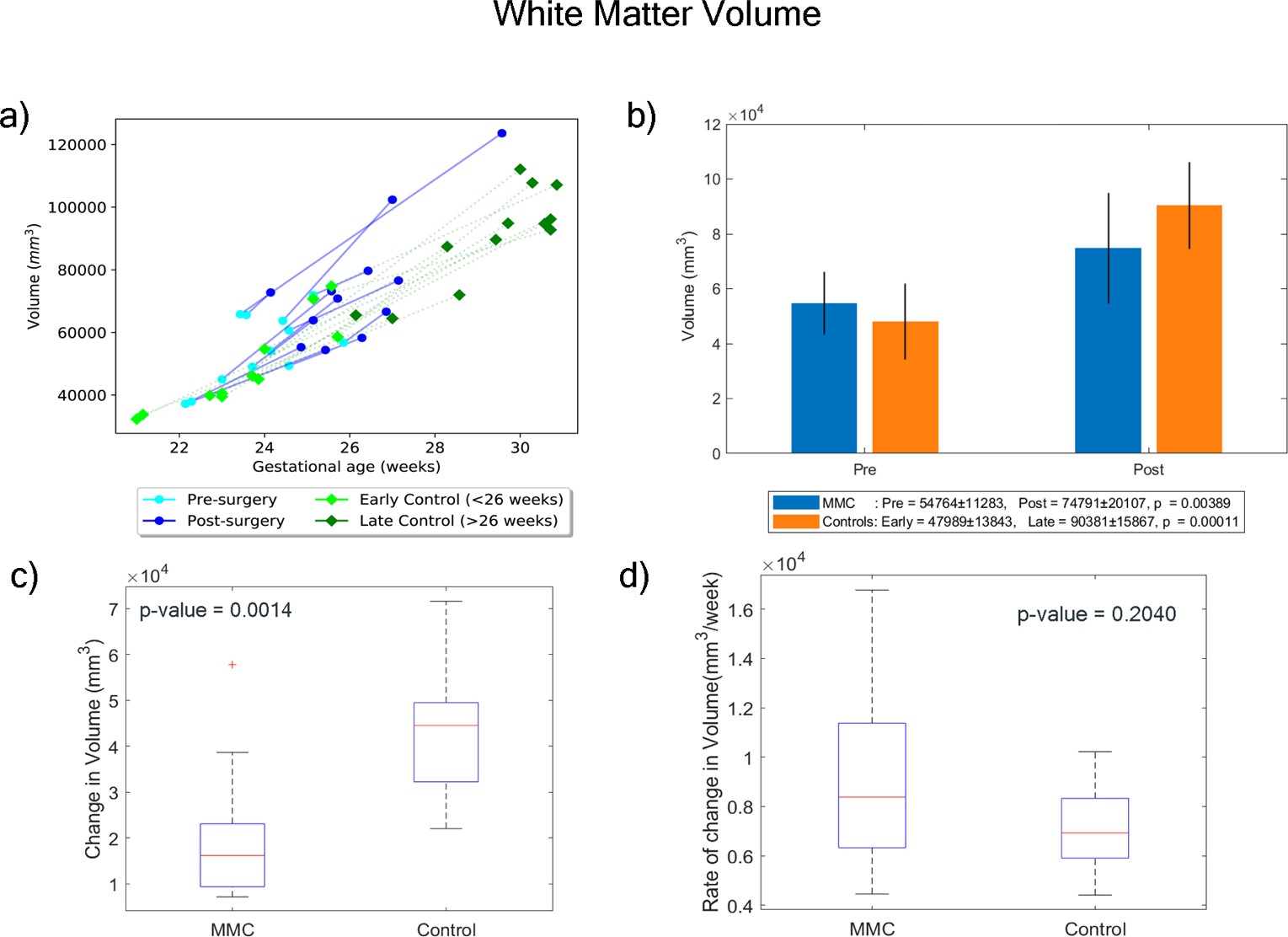


**Fig. 3** (a) Volume (mm^3^) of MMC unmyelinated white matter pre and post-surgery, and age-matched controls against gestational age in weeks (b) Volume (mm^3^) of MMC unmyelinated white matter pre and post-surgery compared to early and late controls. The p values compare differences in measurements between pre and post MMC surgery, and early and late controls (c) Absolute change in volume (mm^3^) of unmyelinated white matter in fetal brain after fetal surgery for MMC compared to control cases (d) Rate of change in volume/time (mm^3^/week) of unmyelinated white matter after surgery for MMC against age-matched controls.

Unmyelinated White Matter Shape Parameter


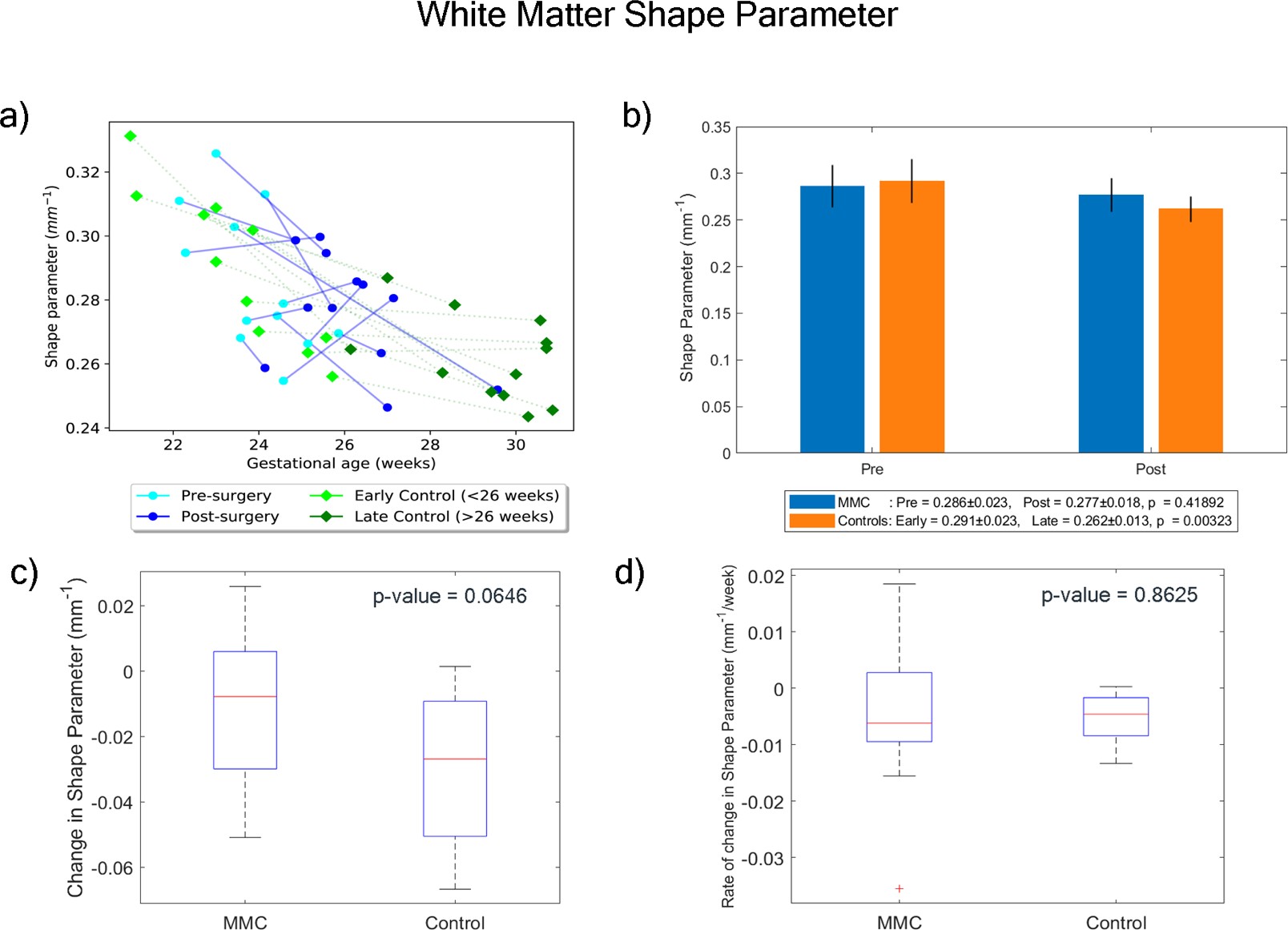


**Fig. 4** (a) Shape parameter (mm^-1^) of MMC unmyelinated white matter pre and post-surgery, and age-matched controls against gestational age in weeks. (b) Shape parameter (mm^-1^) of MMC unmyelinated white matter pre and post-surgery compared to early and late controls. The p values compare differences in measurements between pre and post MMC surgery, and early and late controls to controls (c) Absolute difference in change in Shape parameter (mm^-1^) of unmyelinated white matter after surgery for MMC compared to controls (d) Rate of change in shape parameter/time (mm^-1^/week) of unmyelinated white matter after surgery for MMC against age-matched controls.


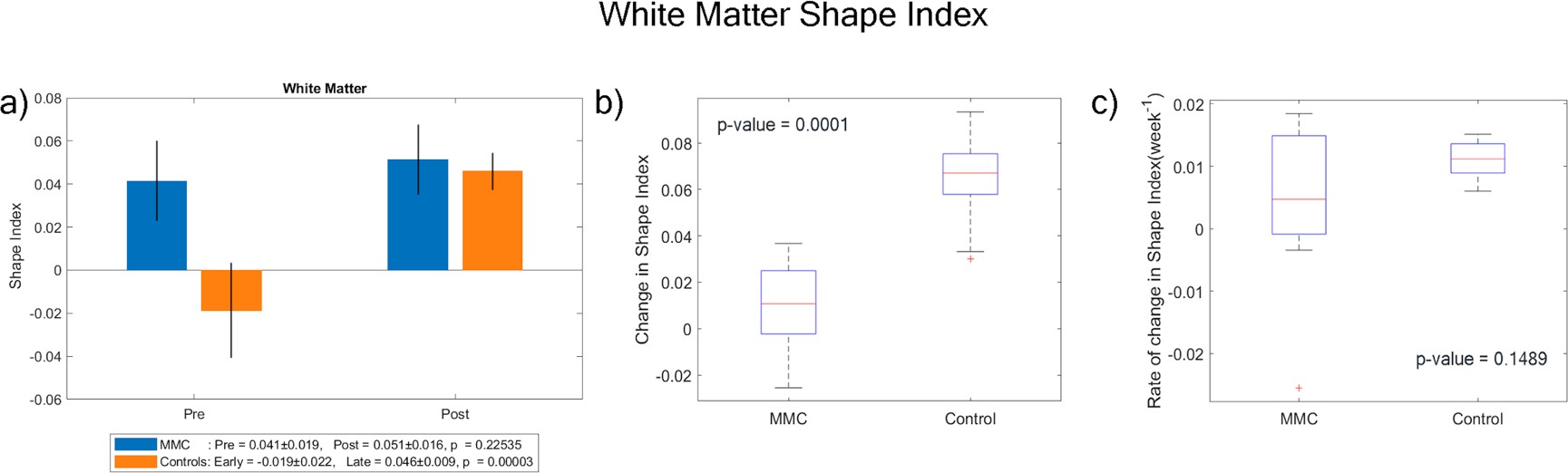


Unmyelinated White Matter Shape Index

**Fig. 5** (a) Shape Index of MMC unmyelinated white matter pre and post-surgery compared to early and late controls. The p values compare differences in measurements between pre and post MMC surgery, and early and late controls to controls (b) Absolute difference of change in unmyelinated white matter after surgery for MMC against age-matched controls (c) Rate of change in shape index/time (week^-1^) of unmyelinated white matter after surgery for MMC against age-matched controls


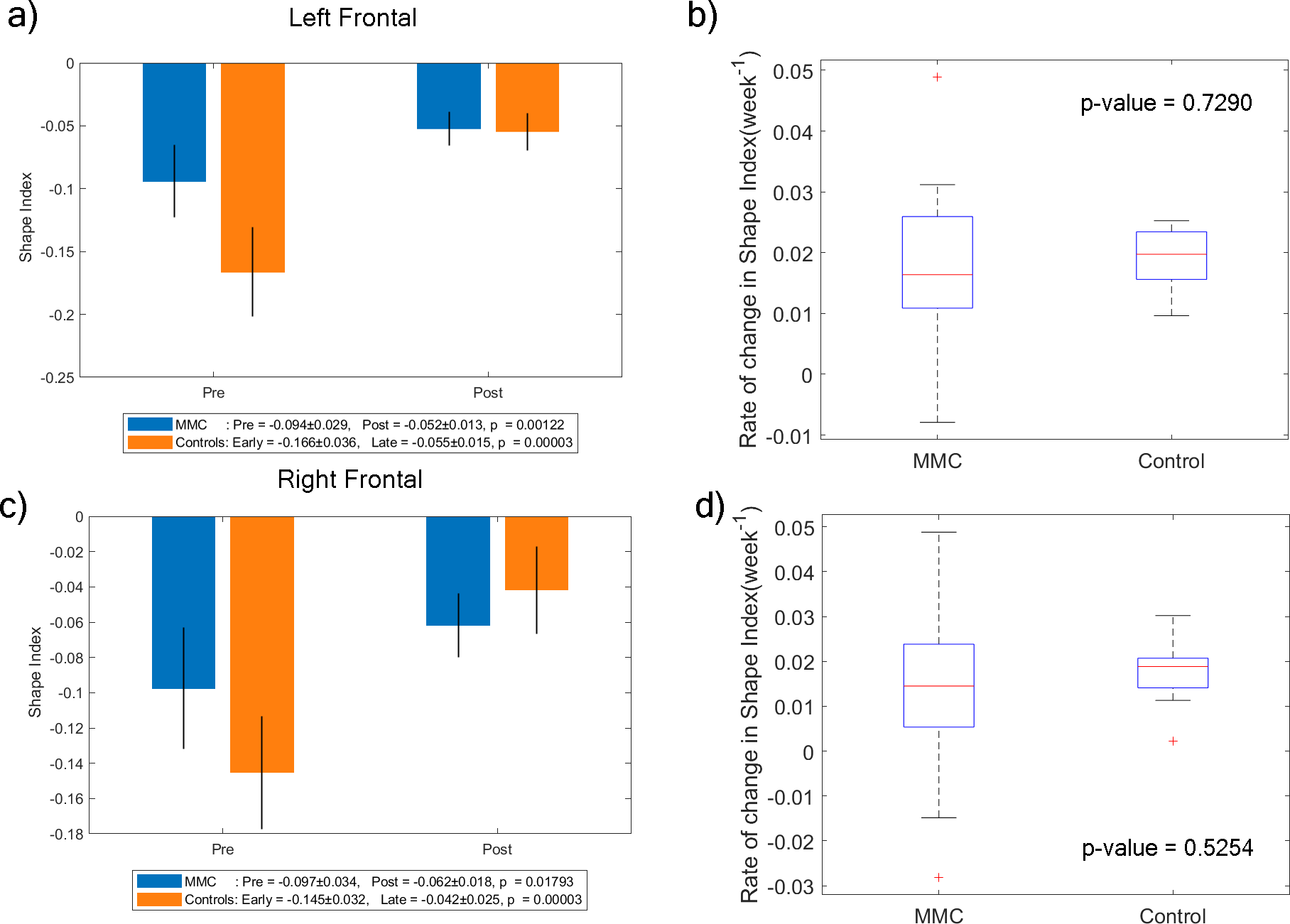


**Fig. 6** (a) Shape Index of MMC left frontal lobe pre and post-surgery compared to early and late controls. The p values compare differences in measurements between pre and post MMC surgery, and early and late controls to controls (b) Rate of change in shape index/time (week^-1^) of left frontal lobe after surgery for MMC against age-matched controls (c) Shape Index of MMC right frontal lobe pre and post-surgery compared to early and late controls. The p values compare differences in measurements between pre and post MMC surgery, and early and late controls to controls (d) Rate of change in shape index/time (week^-1^) of right frontal lobe after surgery for MMC against age-matched controls.


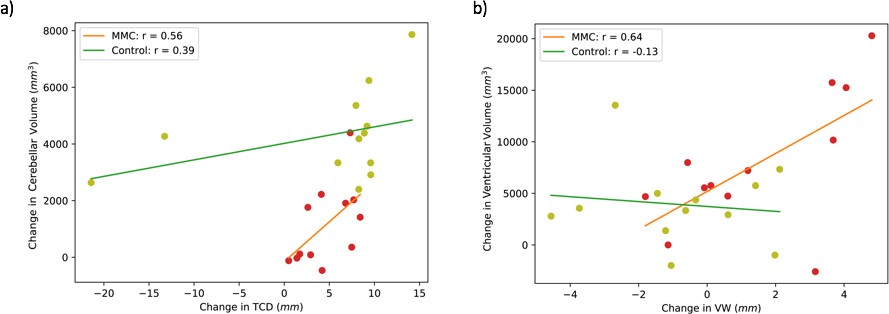


**Fig. 7** Anatomical correlations of (a) TCD against change in cerebellar volume (mm^3^) and (b) VW against change in ventricular volume (mm^3^) after MMC surgery.

# Gestational Age at MRI scans:

Table 1: Gestational Age of Population at the time of MRI

|  | Initial MRI | | P value |
| --- | --- | --- | --- |
|  | Pre-Surgery MMC Cases (n=12) | Early Controls (n=12) | 0.181 |
| Mean GA | 23^+6^ | 23^+2^ |  |
| Range +/- SD | 22^+0^-25^+0^ +/- 1^+0^ | 21^+0^-25^+0^ +/- 1^+2^ |  |
|  | Second MRI | |  |
|  | Post-Surgery MMC Cases (n=12) | Late Controls (n=12) | < 0.001* |
| Mean GA | 26^+1^ | 28^+6^ |  |
| Range +/- SD | 24^+0^-29^+0^ +/- 1^+2^ | 26^+0^-30^+0^ +/- 1^+3^ |  |

jjjj

*statistically significant

†a Early Control case indications for fetal MRI: kidney cyst, trachea-oesophageal fistula, arthrogryposis and micrognathia, vascular liver malformation, placenta praevia, epidermal inclusion cyst, maternal cervical teratoma, urogenital malformation, large choledochus cyst, fetal scoliosis, hyperechogenic lung, gastroschisis

†b Late Control case indications for fetal MRI: enlarged thyroid gland, pelvic ureteric junction obstruction, laryngo-oesophageal fistula, sacrococcygeal teratoma, lymphangioma, abnormally invasive placenta, Liver mass, hyperflexion of both hands, talipes, limb reduction defect
